# Socioeconomic and Demographic Factors Associated with the Influence of the Food Traffic Light Labeling on the Decision of the Adult Population of Ecuador to Purchase Processed Foods, 2018

**DOI:** 10.3390/nu15040885

**Published:** 2023-02-09

**Authors:** Paolo Alfredo Bobbio Gonzáles, Diego Azañedo, Akram Hernández-Vásquez

**Affiliations:** 1 Faculty of Health Sciences, Universidad Científica del Sur, Lima 15067, Peru; 2 Centro de Excelencia en Investigaciones Económicas y Sociales en Salud, Vicerrectorado de Investigación, Universidad San Ignacio de Loyola, Lima 15024, Peru

**Keywords:** socioeconomic factors, nutritional labeling, food labeling, product labeling, Ecuador

## Abstract

To determine the socioeconomic and demographic factors associated with the influence of the nutritional traffic light (NTL) on the decision to purchase processed foods using information from the National Health and Nutrition Survey (ENSANUT) 2018 of Ecuador, a cross-sectional and analytical study based on a secondary analysis of the information from the ENSANUT 2018 was performed. We collected data from 25,932 participants 18 years of age or older who knew or had seen the NTL, and for whom complete information on the variables of interest for the study was available. The “Influence of the NTL on the purchase decision of processed foods” was the outcome variable of the study. Generalized linear models of the Poisson family, with log link, were used to assess the association between socioeconomic factors and outcome, using crude (PR) and adjusted (aPR) prevalence ratios, with 95% confidence intervals (CI) and a *p*-value < 0.05. Participants who understood the NTL (aPR: 2.49; 95% CI: 2.19–2.83), with a higher educational level (aPR: 1.33; 95% CI: 1.09–1.61), women (aPR 1.06; 95% CI: 1.01–1.10), and who had a partner (aPR 1.09; 95% CI: 1.04–1.14) were more likely to be influenced by the NTL when deciding to purchase processed foods, compared to people who did not understand the NTL, who had no educational level or who only attended a literacy center, were men, and those without a partner. The inhabitants of the coastal region (aPR: 0.92; 95% CI: 0.88–0.97), the Amazon (aPR 0.93; 95% CI: 0.88–0.98), and the insular region (aPR 0.76; 95% CI: 0.68–0.84) had few probabilities of being influenced by the NTL in the decision to purchase processed foods, in comparison with the residents of the highlands. Similarly, compared to non-poor people, poor people had a lower probability of being influenced by the NTL (aPR 0.89; 95% CI: 0.82–0.97). Factors associated with the influence of NTL on the decision to purchase processed foods were identified. It is recommended to reformulate and focus awareness strategies for using the NTL to purchase processed foods by taking into account the associated factors.

## 1. Introduction

The last decades have been characterized by an increase in the incidence of overweight adults worldwide. In 2016, the World Health Organization reported that, worldwide, 39% of adults were overweight and 18% were obese [1]. Latin America is no stranger to this problem, since between 2000 and 2016 the prevalence of overweight and obesity in adults increased from 49.6% to 59.5% and from 15.2% to 24.7%, respectively [2]. The figures for Ecuador are similar, with 65% of its population being overweight and between 23.4% and 45.0% of people being obese, according to different estimates [3,4]. This increase in the prevalence of overweight and obesity in the population increases the risk of developing chronic non-communicable diseases (NCDs), such as hypertension, type 2 diabetes mellitus, and cardiometabolic diseases [5,6,7]. In fact, in the year 2021, three of the five leading causes of death in the general population in Ecuador were NCDs, including ischemic heart disease (12.4%), diabetes mellitus (5.3%), and cerebrovascular disease (4.8%) [8].

Due to its rapid increase in most developed and developing countries, obesity is currently considered to be a global epidemic [9]. This condition is related to multiple factors, including sociodemographic, genetic, and behavioral ones, including a sedentary lifestyle, high-calorie intake, and low fiber intake [10]. Thus, in Ecuador, in recent years, a high consumption of processed foods that are dense in calories and with macronutrient and salt content above international dietary recommendations has been described [11,12]. Likewise, between 2000 and 2013, per capita retail sales of ultra-processed food products and beverages in Ecuador increased by 19.8%, with an annual increase of 1.4% [13]. Due to this global problem, several governments have proposed the introduction of a series of strategies to improve the nutritional status of the population. One of these approaches is the nutritional labeling of products, which aims to inform about the nutritional properties of food, as well as the maximum limit of sugar, fat, and salt that consumers should ingest [14,15]. This strategy has been implemented in recent years in most countries of the Americas [14].

One of the most widely used types of labeling, and one that has shown greater acceptability among different population groups [15,16,17], is the nutritional traffic light (NTL) label, which consists of a green, yellow and red color scheme, which indicates a low, medium and high content of the nutritional components of food, mainly sugars, fats and salt [18], respectively. In 2014, the Ecuadorian government, together with the National Agency for Regulation, Control and Sanitary Surveillance (ARCSA), applied the NTL to processed, packaged, and packaged foods [18,19]. However, despite the fact that Ecuadorian users show an adequate understanding of the information provided by the NTL, the attitudes towards its use, as well as the effective use of the traffic light to exercise the decision to purchase a product have not demonstrated much influence in certain segments of the population. In this regard, both the use and the knowledge of the NTL were related to educational and socioeconomic levels [20,21]. Therefore, evidence is required to identify the different socioeconomic factors that could be related to the decision to purchase processed foods through the NTL to provide inputs to reformulate and focus the implementation of nutritional labeling in Ecuador in order to achieve a greater impact on the nutritional status of the population.

Through ENSANUT, the National Institute of Statistics and Censuses (its Spanish acronym is INEC) collects data with the purpose of generating indicators on the main problems and the health situation of the Ecuadorian population [22]. The ENSANUT collected data from the participants who voluntarily agreed to be part of the study, including their sociodemographic characteristics, their understanding of the NTL, and whether or not it influences the decision to purchase food. Using these data, the objective of this study was to determine the socioeconomic and demographic factors associated with the influence of the NTL on the decision to purchase processed foods in the Ecuadorian population.

## 2. Materials and Methods

### 2.1. Study Design and Data Source

An observational, cross-sectional and analytical study was carried out. The freely available database of ENSANUT 2018 was used. This survey is carried out by INEC and its main objective is to generate indicators of the main problems and the health situation and nutrition of the Ecuadorian population in order to evaluate and generate public policies [23].

ENSANUT 2018 is a nationally representative survey performed to update the information collected in 2012. The scope of the geographical coverage of ENSANUT 2018 covers the national, regional and provincial levels. In the ENSANUT 2018 data collection process, teams of previously trained interviewers visited the homes of the selected households and conducted interviews with eligible household members to apply five different questionnaires: those concerning the household, women of childbearing age from 12 to 49 years of age, sexual and reproductive health for men 12 years and older, risk factors in girls and boys 5–18 years of age, and child development for boys and girls under 5 years of age [23].

### 2.2. Population and Sample

The ENSANUT 2018 target population was women from 10 to 49 years of age, children under five years of age, men 12 years of age and over, and girls and boys between 5 and 11 years of age. The ENSANUT 2018 sampling units were the conglomerate (primary sampling unit) and private dwellings (secondary sampling unit). For the selection of the sample, a two-stage and stratified probabilistic sampling method was used that included a total of 2591 conglomerates and 46,638 homes selected nationwide with geographic coverage of the 24 provinces of Ecuador with urban and rural areas. Of the 46,638 homes selected and visited, 43,097 were included (a national coverage of 92.4%) [23].

The subsample for this study consisted of 25,932 people aged 18 or over who knew or had seen the nutritional traffic light, and for whom complete information on the variables of interest for the study were available.

### 2.3. Variables and Measurements

#### 2.3.1. Outcome Variable

The “Influence of the nutritional traffic light on the purchase decision of processed foods” was the outcome variable of the study. This variable was constructed from the question included in the question section on labeling of processed foods and beverages of the “Household” questionnaire: Does the nutritional traffic light influence your decision to purchase food? For the purpose of this study, the variable was recoded as 1 in those who stated that the NTL influenced the decision to purchase processed foods and 0 otherwise.

#### 2.3.2. Independent Variables

The following study covariates were used, which were selected based on previous studies on food labeling and the questions available in the ENSANUT 2018. The following variables were included: age group (18 to 49, 50 to 64, 65 and over) [24], sex (male, female) [25], educational level (none or literacy center, basic education, middle/high school, higher education) [24], marital status (without a partner, with a partner) [26,27], area of residence (urban, rural), geographic region (highlands, coast, Amazon, insular), poverty due to unsatisfied basic needs (not poor, poor) [28,29], ethnicity (non-indigenous, indigenous) [30], and understanding of the nutritional traffic light (no, yes) [24]. Regarding the poverty due to unsatisfied basic needs, in our study a household was classified as poor if it had any of the following household characteristics: high economic dependency, inadequate physical characteristics, overcrowding, without access to basic services, and those with children who do not attend school [31,32].

### 2.4. Statistical Analysis

Data processing and analysis was performed using the Stata 17 statistical program (Stata Corporation, College Station, TX, USA). Similarly, for all of the estimates, the weighting factor and the sample design of the survey were included, the Stata command svy was used, as was the subpop option (adults).

Descriptive and associational analyses were performed. In the descriptive analysis, absolute frequencies and weighted proportions were obtained. Next, a bivariate analysis was performed to assess the association between the proposed covariates and the outcome variable using Pearson’s chi-square tests with Rao-Scott correction. Similarly, to analyze the association between the covariates and the dependent variable, generalized linear models of the Poisson family were used, with a logarithmic link function, in order to obtain crude (PR) and adjusted prevalence ratios (aPR). The adjusted analysis included the independent variables with a *p*-value < 0.20 obtained in the crude analysis. Multicollinearity in the independent variables was also evaluated using the variance inflation factor (VIF), considering VIF values > 10 as multicollinearity (no multicollinearity was found in this study). *p* values < 0.05 were considered statistically significant and confidence intervals were calculated at 95% (95% CI).

### 2.5. Ethical Considerations

This study was approved by the Institutional Research Ethics Committee of the Universidad Científica del Sur under registration code 485-2021-PRE17. Likewise, the present study carried out an analysis of anonymized secondary data that is in the public domain and does not allow the identification of the evaluated participants. The ENSANUT 2018 databases are freely available on the INEC website: https://www.ecuadorencifras.gob.ec/salud-salud-reproductiva-y-nutricion/ (accessed on 20 June 2022).

## 3. Results

Data from a total of 25,932 Ecuadorian adult participants were processed (Table 1). The majority were women (51.6%), from the age group of 18 to 49 years (73.9%), had a partner (62.1%), and had obtained a secondary or high school education (37.2%). Only 5.1% considered themselves Indigenous. Most of the participants lived on the coast (50.8%), and in an urban area (76.8%). Likewise, 91.3% were not poor, according to their unsatisfied basic needs, and 89.1% of the participants stated that they understood the NTL in processed foods (Table 1).

The influence of the NTL on the decision to purchase processed foods occurred in a greater proportion in those who stated that they understood the NTL (56.2%), residents of urban areas (53.9%), participants with a higher educational level (61.4%), non-indigenous participants (52.6%), residents of the highlands (55.5%), women (53.7%), non-poor people (53.4%), and people with a partner (53.4%). Only age did not present a significant association with the influence of the NTL on the decision to purchase processed foods (Table 2).

In the multivariate analysis, participants who understood the NTL were more likely to be influenced by this strategy when deciding to purchase processed foods (aPR: 2.49; 95% CI: 2.19–2.83) (Table 3). Similarly, those who had a higher educational level were more likely (aPR: 1.33; 95% CI: 1.09–1.61) to be influenced by the NTL compared to those with no educational level or who had only attended a literacy center. Residents of the coastal region (aPR: 0.92; 95% CI: 0.88–0.97), the Amazon (aPR 0.93; 95% CI: 0.88–0.98) and the insular region (aPR 0.76; 95% CI: 0.68–0.84) had a lower probability of being influenced by the NTL in the decision to purchase processed foods compared to residents of the highland region. Women presented a higher probability (aPR 1.06; 95% CI: 1.01–1.10) of being influenced by the NTL in purchase decisions than men. Likewise, poor people compared to non-poor people had a lower probability of purchases being influenced by the NTL (aPR 0.89; 95% CI: 0.82–0.97). Finally, compared to people without a partner, those with a partner had a higher probability (aPR 1.09; 95% CI: 1.04–1.14) of being influenced by the NTL in their decisions to purchase processed foods.

## 4. Discussion

The present study aimed to determine the socioeconomic and demographic factors associated with the influence of the NTL on the decision to purchase processed foods using the information collected by the ENSANUT in 2018. Approximately 9 out of 10 survey participants stated that they understood the NTL. In addition, of these, 56.2% said they felt influenced by the traffic light when choosing processed foods. Participants who understood the NTL had a higher educational level, were female, had a partner and were more likely to be influenced by nutritional labeling when buying processed food compared to those who did not understand the NTL label, had no education or who had only attended a literacy center, men, and those who did not have a partner, respectively. On the other hand, residents of the coastal, Amazon, or insular regions, and those living in a state of poverty presented a lower probability of being influenced by the NTL compared to residents of the highlands, and those who were not poor. It is necessary to propose and evaluate the effectiveness of new interventions that take into account the characteristics associated with the influence of the NTL on food purchases, and that seek to increase awareness in the population about the importance of using labeling in their food decisions.

People who understood the NTL were more likely to be influenced by the labeling when choosing processed foods compared to those who did not understand it. In this regard, studies in subpopulations of Ecuador found that although the population understands the information provided by the traffic light, it is frequently not used in the decision to purchase processed foods [20,21]. A qualitative study in the general population of five provinces of Ecuador found that despite the majority of study participants understanding the NTL, it was very infrequently used by children and adult men to make purchasing decisions. On the other hand, adolescents interested in taking care of their health and adult women used it more frequently [21]. However, a study carried out in 2015 on 73 consumers who went to two supermarkets in Quito, identified that greater knowledge about traffic light labeling was associated with its effective use to make food purchases. However, 9 out of 10 people who were aware of the traffic light had a high educational level, which is to be expected in urban areas of Ecuador [20]. In addition, it should be noted that these studies present limited representativeness because they included subpopulations of Ecuador. Furthermore, they were carried out between 2015 and 2016, and by the time of the ENSANUT 2018 the population may have had greater exposure to awareness campaigns with regard to the NTL. Thus, within its first year of implementation in Ecuador, the traffic light failed to reduce the consumption of carbonated beverages with high sugar content, with higher consumption in homes with a low socioeconomic level. [11]. In this sense, certain characteristics at the population level, such as age, sex, area of residence, and educational and socioeconomic level, could be determinants of the effective use of the NTL to make food purchase decisions, despite having knowledge of this program. Likewise, interest in healthy eating can be an important element in making informed purchasing decisions; therefore, greater awareness of health care among the population is required.

People with a higher educational level were more likely to be influenced by nutritional labeling when buying processed foods. This is consistent with what has been reported by other studies in the literature. A study on older adults in the Netherlands identified that those with a medium to a higher level of education had greater knowledge about the importance of proper food selection, as well as of the impact and repercussions of the prolonged consumption of processed foods on health [28]. In the same way, another study that used information from 12 European countries identified that a high educational level was associated with more adequate nutritional consumption, even in countries with a low gross domestic product, describing a mitigating role of education in the diet in these countries [33]. Therefore, strategies to raise awareness of the use of the NTL should be focused on populations that have a higher proportion of people with a low educational level.

Compared to non-poor people, poor people were less likely to be influenced by the NTL when shopping for processed foods. In The Netherlands, a study reported that participants with a high socioeconomic level gave a high level of importance to the healthiness of a food product above other attributes such as taste, preparation time, product purchase time, and price, compared to those of a lower socioeconomic level, who presented a greater tendency to consume diets high in fat and low in micronutrients, as well as having a lower consumption of fruits and vegetables [28]. Another study, carried out in Luxembourg, identified that being below the poverty line was associated with lower compliance with national nutritional recommendations and with a greater selection of unhealthy foods [29]. In this regard, there is evidence that healthy food options tend to be more expensive compared to unhealthy options [34]; however, this pattern can be context-dependent [35]. Following the implementation of the NTL in Ecuador, it has been reported that households with low-income levels tend to spend more and consume more calories from carbonated soft drinks than households with high socioeconomic levels [11]. Therefore, it is likely that among people with fewer economic resources, the price of food can be a more important deciding factor than nutritional components when buying food.

Regarding the natural regions, residents of the coastal, Amazon, and insular regions showed a lower probability of being influenced by the NTL when deciding to purchase processed food compared to the residents of the highlands. Due to the particular political-administrative division in Ecuador, these results are probably not comparable with other studies. In this regard, the Ecuadorian highlands have important and populated cities with significant levels of economic development, such as Quito, the capital, in which a population with a higher educational level resides, and who reported a greater amount of influence concerning labeling on their food choices. In addition, the information campaigns for the implementation of labeling could have a greater reach in the capital of Ecuador and, therefore, the population may be more aware than in other areas. Similarly, the differences identified could be related to a greater concentration of low-income neighborhoods in the coastal, Amazonian, and insular regions, which tend to offer greater access to food sources that promote inadequate eating patterns [36]. Furthermore, an analysis of eating patterns on the coast and highlands of Ecuador showed that healthier foods are consumed in the highlands than on the coast [37]. Further studies are needed to carry out a more in-depth analysis of the mechanisms of the association identified.

Compared to people without a partner, those with one were more likely to be influenced by the traffic light when buying processed foods. In the United States, a study of workers from two Universities in Southern California reported that married women had healthy consumption patterns, with higher frequencies of consumption of vegetables and diet drinks compared to never-married women, who consumed more sugary drinks [26]. Although no comparable studies were identified, other studies have reported a positive association between marriage and higher fruit and vegetable consumption in older women and men [27,38]. This particular pattern of eating could be explained in that in people with a partner, the responsibilities of acquiring food can be shared, or because the spouses felt responsible for feeding their partners, and thereby reduced impulse purchases and discussed food purchase decisions as a couple [39].

Compared to men, women were more likely to be influenced by the NTL in the decision to purchase foods with traffic light labels. Similar results were observed in a study carried out in the seventh district of Quito, and it was concluded that women use labeling more frequently than men do [25]. Another qualitative study concluded that the responsibility for the selection and preparation of food generally falls on mothers, with little participation from the fathers, thereby highlighting the female role in the selection of food at home [24]. These findings are important since women could assume a fundamental role in raising awareness about the use of the NTL in food purchasing decisions in different contexts, such as family and work.

Among the main limitations of this study is its cross-sectional design, which does not allow for the establishing of causality because there is no temporal relationship between the factors evaluated and the outcome. Likewise, there is the possibility of measurement bias in the recording of the data of the variables studied due to errors on the part of the interviewers or misunderstanding on the part of the participants about the questions formulated. In addition, there could be a social desirability bias, especially for the questions about understanding the NTL and the outcome variable: the participants may have answered yes, even though they do not understand it or are not influenced by it. When buying processed foods, the possibility of this bias can also be affected by variables such as educational level, ethnicity, and whether or not the participant has a partner. In the same way, the focus of this study is exploratory and does not pursue the estimation of the direct association between the different variables evaluated and the outcome of interest. In this regard, the multiple tests of hypotheses carried out may increase the possibility of committing Type I errors; however, carrying out the study is justified because the outcome of interest and possible related factors have been scarcely studied in the local and international literature. Therefore, future research should take into account the study of individual factors while accounting for longitudinal designs within causal schemes that reduce the possibility of confusion and overfitting. Finally, as it is a secondary analysis, only the variables found in the survey were included in the study, and other factors such as nutritional status, chronic disease, and acute disease may have been involved.

## 5. Conclusions

In conclusion, participants who understood the NTL, had a higher educational level, were female, and had a partner had a higher probability of being influenced by the NTL when buying processed food compared to those who did not understand the NTL, had no educational level or who had only attended a literacy center, were men, and who did not have a partner. In addition, residents of the coastal, Amazon, or insular regions and those living in a state of poverty presented a lower probability of being influenced by the NTL in comparison with residents of the highlands and those who were not poor. It is recommended that the Ecuadorian government reformulate and focus awareness strategies on the use of the NTL in the decision to purchase processed foods, taking into account its associated factors, in order to obtain a greater scope and effectiveness of this measure. Similarly, the effectiveness of the application of nutrition labeling must be evaluated over time in order to make informed decisions in a timely manner. Furthermore, it is necessary for the national regulatory entities to monitor compliance with the placement of the NTL on the front of the products according to the existing implementation law in order for the labels to be more visible to users.

## Figures and Tables

**Table 1:** Characteristics of the adult population of Ecuador included in the ENSANUT, 2018.

Characteristic	Absolute Frequency (n = 25,932)	Weighted Proportion *
Understanding of the NTL		
No	2899	10.9
Yes	23,033	89.1
Place of residence		
Urban	17,956	76.8
Rural	7976	23.2
Age group		
18–49	20,077	73.9
50–64	4045	18.2
64 or more	1810	7.9
Education level		
None or Literacy Center	431	1.5
Basic education	8903	34.5
Middle school/high school	10,032	37.2
Superior	6566	26.7
Ethnicity		
Non-indigenous	23,713	94.9
Indigenous	2219	5.1
Natural region		
Highlands	10,037	45.0
Coast	9435	50.8
Amazon	5136	4.1
Insular	1324	0.1
Sex		
Men	12,087	48.4
Women	13,845	51.6
Poverty by UBN		
Not poor	23,141	91.3
Poor	2791	8.7
Marital status		
With a partner	9164	37.9
Without a partner	16,768	62.1

* The weighting factor and sample specifications of the ENSANUT 2018 were included. UBN: Unsatisfied Basic Needs; NTL: Nutritional Traffic Light.

**Table 2:** Characteristics associated with the influence of the nutritional traffic light on the decision to purchase processed foods in the adult population of Ecuador, 2018.

Characteristics	Does Not Influence % (95% CI)	Influences % (95% CI)	*p*-Value *
Overall	47.6 (46.3–48.9)	52.4 (51.1–53.7)	
Understanding of the NTL			
No	78.7 (75.8–81.3)	21.3 (18.7–24.2)	<0.001
Yes	43.8 (42.5–45.2)	56.2 (54.8–57.5)	
Place of residence			
Urban	46.1 (44.6–47.7)	53.9 (52.3–55.4)	<0.001
Rural	52.6 (50.3–54.9)	47.4 (45.1–49.7)	
Age group			
18–49	48.0 (46.6–49.5)	52.0 (50.5–53.4)	0.484
50–64	46.4 (43.6–49.2)	53.6 (50.8–56.4)	
64 or more	46.8 (43.1–50.5)	53.2 (49.5–56.9)	
Education level			
None or Literacy Center	64.2 (57.1–70.8)	35.8 (29.2–42.9)	<0.001
Basic education	53.0 (51.0–54.9)	47.0 (45.1–49.0)	
Middle school/high school	48.5 (46.4–50.7)	51.5 (49.3–53.6)	
Superior	38.6 (36.2–41.0)	61.4 (59.0–63.8)	
Ethnicity			
Non-indigenous	47.4 (46.0–48.7)	52.6 (51.3–54.0)	0.017
Indigenous	52.9 (48.4–57.3)	47.1 (42.7–51.6)	
Natural region			
Highlands	44.5 (42.6–46.3)	55.5 (53.7–57.4)	<0.001
Coast	50.1 (48.2–52.1)	49.9 (47.9–51.8)	
Amazon	51.4 (49.3–53.5)	48.6 (46.5–50.7)	
Insular	56.2 (51.5–60.7)	43.8 (39.3–48.5)	
Sex			
Men	49.1 (47.2–50.9)	50.9 (49.1–52.8)	0.017
Women	46.3 (44.7–47.9)	53.7 (52.1–55.3)	
Poverty by UBN			
Not poor	46.6 (45.2–47.9)	53.4 (52.1–54.8)	<0.001
Poor	58.9 (55.5–62.3)	41.1 (37.7–44.5)	
Marital status			
With a partner	49.3 (47.3–51.4)	50.7 (48.6–52.7)	0.022
Without a partner	46.6 (45.1–48.1)	53.4 (51.9–54.9)	

The weighting factor and sample specifications of the ENSANUT 2018 were included. Values are presented as % of the row. * The *p*-value was calculated using the chi-square test with Rao-Scott correction. UBN: Unsatisfied Basic Needs; NTL: Nutritional Traffic Light.

**Table 3:** Factors associated with the influence of food traffic light labeling on the decision to purchase processed foods in the adult population of Ecuador, 2018.

Characteristics	Crude Model		Adjusted Model *	
	PR (95% CI)	*p*-Value	aPR (95% CI)	*p*-Value
Understanding of the NTL				
No	Reference		Reference	
Yes	2.63 (2.32–2.99)	<0.001	2.49 (2.19–2.83)	<0.001
Place of residence				
Urban	Reference		Reference	
Rural	0.88 (0.83–0.93)	<0.001	0.99 (0.93–1.05)	0.688
Age group				
18–49	Reference		Not included	
50–64	1.03 (0.97–1.09)	0.287		
64 or more	1.02 (0.95–1.10)	0.530		
Education level				
None or Literacy Center	Reference		Reference	
Basic education	1.32 (1.08–1.60)	0.006	1.12 (0.93–1.35)	0.222
Middle school/high school	1.44 (1.18–1.75)	<0.001	1.16 (0.96–1.40)	0.125
Superior	1.72 (1.41–2.09)	<0.001	1.33 (1.09–1.61)	0.004
Ethnicity				
Non-indigenous	Reference		Reference	
Indigenous	0.90 (0.81–0.98)	0.023	0.95 (0.87–1.05)	0.313
Natural region				
Highlands	Reference		Reference	
Coast	0.90 (0.85–0.95)	<0.001	0.92 (0.88–0.97)	0.002
Amazon	0.87 (0.83–0.92)	<0.001	0.93 (0.88–0.98)	0.009
Insular	0.79 (0.71–0.88)	<0.001	0.76 (0.68–0.84)	<0.001
Sex				
Men	Reference		Reference	
Women	1.06 (1.01–1.10)	0.017	1.06 (1.01–1.10)	0.011
Poverty by UBN				
Not poor	Reference		Reference	
Poor	0.77 (0.71–0.84)	<0.001	0.89 (0.82–0.97)	0.007
Marital status				
With a partner	Reference		Reference	
Without a partner	1.05 (1.01–1.10)	0.023	1.09 (1.04–1.14)	<0.001

The weighting factor and sample specifications of the ENSANUT 2018 were included. PR: Prevalence Ratio, aRP: Adjusted Prevalence Ratio. * Adjusted for the variables shown in the column. UBN: Unsatisfied Basic Needs; NTL: Nutritional Traffic Light.

## Data Availability

The ENSANUT 2018 databases are freely available on the INEC website: https://www.ecuadorencifras.gob.ec/salud-salud-reproductiva-y-nutricion/ (accessed on 20 June 2022).
